# Primary Leiomyoma of the pleura

**DOI:** 10.1186/1477-7819-9-76

**Published:** 2011-07-14

**Authors:** Xiaoming Qiu, Daxin Zhu, Sen Wei, Gang Chen, Jun Chen, Qinghua Zhou

**Affiliations:** 1 Department of Lung Cancer Surgery, Tianjin Key Laboratory of Lung Cancer Metastasis and Tumor Microenvironment, Tianjin Lung Cancer Institute, Tianjin Medical University General Hospital, Tianjin 300052, China

**Keywords:** Pleural tumor, Leiomyoma, Smooth muscle tumor

## Abstract

Primary leiomyoma of the pleura is extremely rare. A 45-year-old man presented with a complaint of right chest pain. Chest computed tomography demonstrated a solid, round pleural mass in the right anterior chest wall. The mass was completely resected, and histopathological examination revealed a localized primary pleural leiomyoma. The patient was followed and has been disease-free for over 15 months. This is the first report of primary leiomyoma of the pleura in China. A review of the literature on primary leiomyoma of the pleura is presented.

## Introduction

Leiomyomas are benign smooth muscle tumors that are commonly encountered in the urogenital tract, occasionally in the gastrointestinal tract, and rarely in the respiratory tract [1]. However, leiomyomas originating from the pleura are extremely rare. To our knowledge, only 8 cases have been published up to the present time. Here, we present the first primary pleural leiomyoma in China.

## Case report

**Figure 1:**
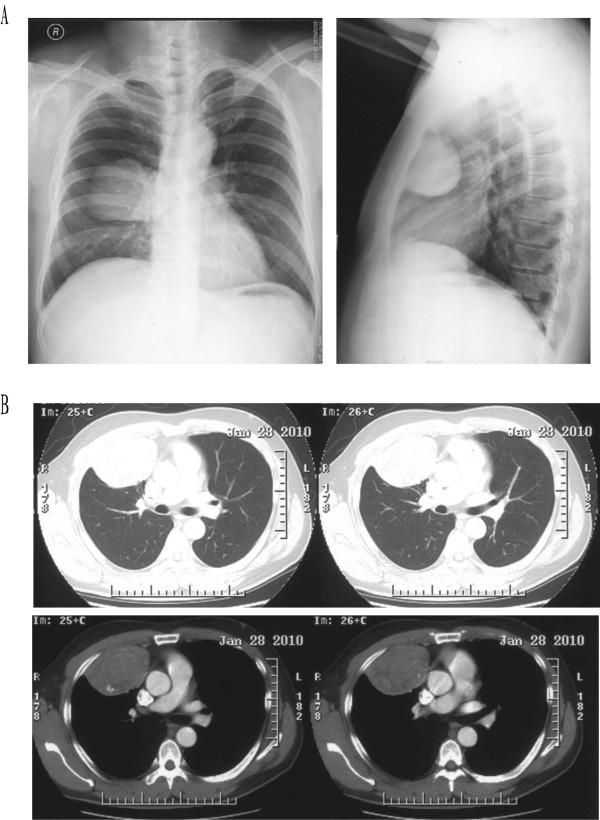
**Chest images** (A) Chest radiograph. A mass is seen in the right middle lung field on chest roentgenogram; (B) Chest contrast enhanced computed tomography scans. A solid, round pleural mass is seen in the right anterior chest wall, with heterogeneous density and calcification. It is well capsulated and compresses the lung parenchyma without any sign of infiltration of lung or chest wall.

A 45-year-old man without any significant past history was admitted to our department complaining of right chest pain persisting for 3 days. A mass was seen in the right middle lung field on chest roentgenogram (Figure 1A). However, physical examination was unremarkable. Computed tomography showed a solid, round pleural mass measuring 5.9 cm × 8 cm × 6.2 cm located in the right anterior chest wall, with " heterogeneous density and calcification. It was well capsulated and compressed the lung parenchyma without any sign of infiltration of the lung or chest wall (Figure 1B).

**Figure 2:**
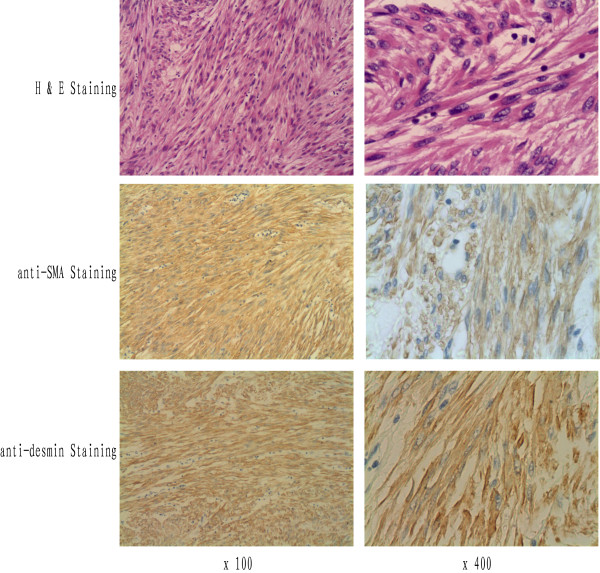
**Hematoxylin-and-eosin staining reveals a proliferation of interlaced fascicles of spindle cells showing moderate atypia and less than 1/50 mitotic figures per high power field**. Immunohistochemical staining reveals diffuse and strongly positive reactions with both anti-SMA and -desmin antibodies.

The patient underwent a right posterolateral thoracotomy. A giant, well-capsulated smooth-surfaced tumor was observed in the anterior chest wall. It was easily resected en-bloc with surrounding tissue. Grossly, the tumor measured 9 cm × 6 cm × 5 cm and was covered with pleura. The cut tumor was solid white with cystic cavities. Pathological examination revealed a proliferation of interlaced fascicles of spindle cells showing moderate atypia and less than 1 mitotic figure per 50 high power fields (Figure 2). Immunohistochemistry revealed diffuse and strong staining for both smooth muscle actin (SMA) and desmin (Figure 2). Tumor tissue stained negative for estrogen receptor, progesterone receptor, CD117, and HMB45, and was weakly positive for S-100 protein (data not shown).

The patient had an uneventful recovery, and there has been no sign of recurrence 15 months after surgery. Since the malignant potential of the tumor could not be determined, close follow-up of the patient was advised.

## Discussion

Primary intrathoracic soft tissue tumors are unusual. They can originate from the upper or lower respiratory tract or from the mediastinum. The differential diagnosis for pleural spindle cell neoplasms includes solitary fibrous tumor, smooth muscle tumor, spindle cell carcinoma, thymoma, sarcomatoid variant of mesothelioma, and leiomyoid variant of mesothelioma [2-5]. Leiomyomas commonly originating from urogenital tract and gastrointestinal tract are most benign smooth muscle tumors, and rarely originates from respiratory tract and pleura. So far, there were only 8 primary pleural leiomyomas reported in the world [1,6-11]. Table 1 summarizes patient and tumor characteristics of the 8 previously described cases and our case. Of these cases, 6 were asymptomatic and 3 had nonspecific chest pain related to tumor size and location. The tumor appears to occur more frequently in young to middle-aged females (6 of nine cases).

**Table 1:** Clinical features of published case reports of primary pleural leiomyoma

Case	Author	Gender/age	Clinical manifestations	Clinical course	Tumor size (cm)	Follow-up after operation
1	Tanaka T et al [6]	F/40	Asymptomatic	Complete resection	3,5 × 3,0	17 months Alive without recurrence

2	Moran CA et al [7]	F/21	Asymptomatic, large left pleural tumor involving diaphragm	Too large for complete resection	Multiple fragments of firm, grey tissues	4 months Alive

3	Moran CA et al [7]	F/23	Asymptomatic, large mass arising from the right pleura	Too large for complete resection	10.0 × 9.0 × 5.5	6 months Alive

4	Proca DM et al [1]	M/32	Asymptomatic	Resected 4 years after needle biopsy, when enlarged and invading the chest wall	4.3 × 7.0	12 months. Alive without recurrence

5	Mochizuki H et al [8]	M/33	Asymptomatic	Complete resection with VATS	3 × 2	Unknown

6	Nose N et al [9]	F/55	Asymptomatic	Complete resection with VATS	1.5 × 1.5	26 months Alive without recurrence

7	Turhan K et al [10]	F/50	Chest pain	Complete resection	4.0 × 4.0	53 months Alive without recurrence

8	Rodriguez, PM [11]	F/48	Chest pain	Complete resection	18 × 14 × 11	18 months Alive without recurrence

9	Our case	M/45	Chest pain	Complete resection	9 × 6 × 5	15 months Alive without recurrence

VATS, video-assisted thoracic surgery.

Although this leiomyoma of the pleura appeared benign, with a smooth, well-capsulated surface and unremarkable histologic findings, it has a low but definite malignant potential. Pleural leiomyomas may increase in size with local invasion to the mediastinum and may not be possible to resect completely [7]. They may even metastasize or disseminate through the needle tract years after a transthoracic fine-needle biopsy [1]. Computed tomography-guided transthoracic fine-needle aspiration (FNA) of chest wall tumors is preferred by oncologists, in order to obtain an accurate preoperative tissue diagnosis by histopathological examination [12,13]. Although FNA can be easily performed for most chest tumors, with minimal damage to the primary tumor and low rates of complications, to avoid needle seeding, we believe that FNA should not be performed if the tumor can be resected completely. It should only be performed when the tumor is unresectable or the patient refuses surgery, in order to allow pathologic diagnosis for further medical treatment.

Most tumors can be completely resected if the contrast-enhanced chest CT scan shows a well-capsulated pleural mass with minimal invasion of the adjacent organs, and the patient does not have signs of invasion, such as very severe pain. In general, most primary pleural leiomyomas can be easily and completely resected. If the tumor is small and localized, complete resection can be achieved with minimally invasive surgery such as video-assisted thoracic surgery (VATS) [8,9,14]. In addition, the prognosis is fairly good for patients in whom the tumor is completely resected. The presence of smooth muscle fibers without signs of malignancy (pleomorphism, mitotic figures, and poor differentiation) can be confirmed by hematoxylin and eosin (H&E) staining. Immunohistochemical staining should be positive for SMA, vimentin, desmin, and HH35 soft muscle protein [1,13]. The histological features of H&E-stained tissue sections plus positive staining for SMA and desmin provide unambiguous evidence for the diagnosis of primary pleural leiomyoma.

## Conclusion

In summary, because primary pleural leiomyoma is an extremely rare tumor with low malignant potential, complete resection and close follow-up is advised. Transthoracic fine-needle biopsy is contraindicated if it is possible that the tumor can be completely resected.

## Consent

Written informed consent was obtained from the patient for publication of this Case report and any accompanying images. A copy of the written consent is available for review by the Editor-in-Chief of this journal.

## Competing interests

The authors declare that they have no competing interests.

## Authors' contributions

XQ, DZ, JC and QZ did the surgery, SW and GC collected the data. XQ, JC and QZ wrote the article. All authors read and approved the final manuscript.
